# Supplementary material for: Lower IQ and poorer cognitive profiles in treated perinatally HIV-infected children is irrespective of having a background of international adoption
Source: PLoS One. 2019 Dec 5;14(12):e0224930. doi: 10.1371/journal.pone.0224930 (PMC6894817; doi:10.1371/journal.pone.0224930)
Supplement: S1 Table — Abbreviations: IQ, intelligence quotient, WISC, Wechsler Intelligence Scale for Children; WAIS, Wechsler Adult Intelligence Scale. *We obtained domain scores on learning ability and executive functioning by averaging the subtest scores. (DOCX) [file pone.0224930.s001.docx]

**Supporting Information**

| **Domain outcome** | **Tool** | **Subtest** |
| --- | --- | --- |
| **IQ** | WISC/WAIS-III | Vocabulary |
|  | WISC/WAIS-III | Arithmetic |
|  | WISC/WAIS-III | Block design |
|  | WISC/WAIS-III | Picture arrangement |
| **Executive functioning*** | Trail Making Test | Trail Making Test part A |
|  | Trail Making Test | Trail Making Test part B |
| **Processing speed** | WISC/WAIS-III | Coding |
|  | WISC/WAIS-III | Symbol Search |
| **Working memory** | WISC/WAIS-III | Digit span |
| **Learning ability*** | Rey Auditory Verbal Learning Test | Immediate recall (sum of trials 1-5) |
|  | Rey Auditory Verbal Learning Test | Delayed recall |
| **Visual-motor function** | Beery-Buktenica Developmental Test of Visual-Motor Integration | Beery-Buktenica Developmental Test of Visual-Motor Integration |

**S1 Table: Neuropsychological assessment**
